# Equality in Recipients of Nephrology Awards from International Societies

**DOI:** 10.1016/j.xkme.2022.100505

**Published:** 2022-06-24

**Authors:** Michael Haidinger, Svenja Ravioli, Gregor Lindner

**Affiliations:** 1 Department of Internal and Emergency Medicine, Buergerspital Solothurn, Switzerland; 2 Department of Emergency Medicine, Inselspital, University Hospital Bern, Switzerland

To the Editor:

Evidence of a significant gender gap throughout medicine is increasing. Concerning members and presidents of national societies, women were underrepresented, for example, in internal medicine and intensive care.^1^^,^^2^ Additionally, women were underrepresented in editorial boards of scientific emergency medicine journals and among speakers at large conferences.^3^^,^^4^ Female emergency physicians were underrepresented in leading positions, worked more hours, and were paid less than their male colleagues.^5^

For nephrology, gender distribution in boards of national nephrology societies was more balanced compared to other medical specialities.^6^ Contrarily, a study evaluating proportions of women as chairs, moderators, and speakers at the Kidney Week of the American Society of Nephrology (ASN) showed that women were less often nominated as speakers, moderators, or awardees.^7^ The aim of this study was to evaluate gender distribution in recipients of awards of 3 large international nephrology societies. Naturally, the term “gender” includes more than 2 entities, but for reasons of simplicity “man” and “woman” were chosen to perform this analysis. Since all data was publicly available, need for informed consent or approval by the local ethics committee were waived.

Data concerning awards were collected from the following societies: European Renal Association-European Dialysis and Transplantation Association (www.era-online.org), ASN (www.asn-online.org), and the International Society of Nephrology (www.theisn.org). Data were gathered either from the websites of the respective societies or by direct request. The sex of the recipients was identified by first name, using web-based research and gender identifier software (www.genderchecker.com) in ambiguous cases.

In total, 265 awardees were analyzed from 2011 to 2021. Fifty-three (20%) of the award recipients were women. There were fewer female recipients of European Renal Association-European Dialysis and Transplantation Association awards (15%) than of ASN awards (33%). In the past 3 years, from 2019 to 2021, women have comprised the majority of award winners, representing a substantial increase, with more women than men receiving awards in 2020 and 2021 (Fig 1). An analysis of ASN alone showed that during the last 2 years of the study period, female awardees outnumbered male awardees, indicating that change is in progress (female award winners in 2020: 62%, 2021: 67%). Interestingly, in the United States, the percentage of female nephrologists has been rising, and there are now more female chairs, moderators, and speakers at the ASN annual Nephrology Kidney Week.^8^

**Figure 1:**
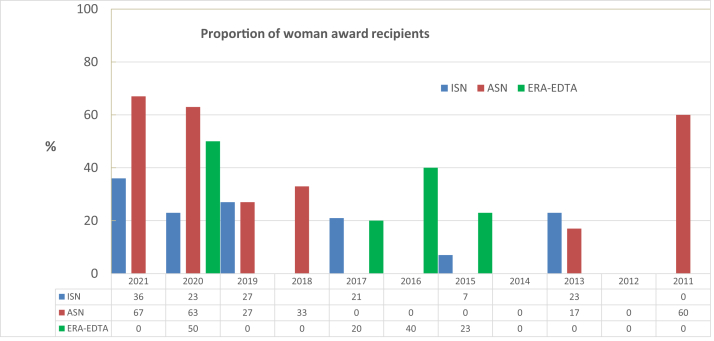
Proportion of female recipients of awards from the respective international society between 2021 and 2011. ISN, International Society of Nephrology.

We also focused on special award categories. ASN, for example, awards 2 different categories; although lifetime awards have been granted for more than 10 years, midcareer awards have been awarded for only the last 3 years. The ASN ‘Lifetime Achievement’ was awarded 52 times between 2000 and 2010 and only 4 (8%) recipients were women (Fig 2). Before 2000, only 3 out of 81 (3.7%) of all prize winners were women (Dr Reneé Habib, Dr Marilyn Farguhar, and Dr Priscilla Kincaid-Smith).

**Figure 2:**
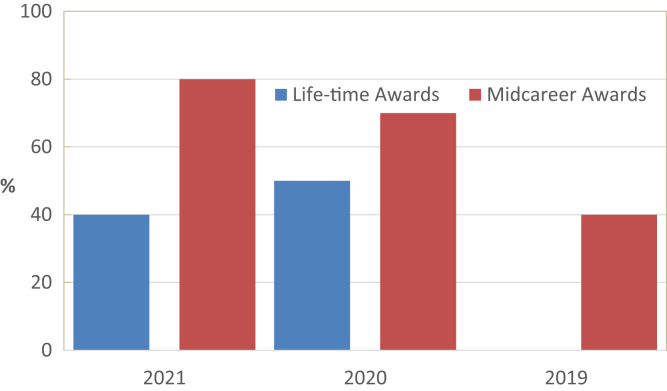
Proportion of female recipients of American Society of Nephrology (ASN) awards between 2021 and 2000.

The International Society of Nephrology grants “Pioneer Awards” to honor physicians working in nephrology who have carried out extraordinary efforts to advance nephrology in a specific country or geographic region. Remarkably, during the study period, Pioneer Awards for Africa, Latin America, and North and East Asia were exclusively received by men.

The present findings show that women were underrepresented as awardees in nephrology, in line with previous studies; an analysis of awardees of the American Urological Association since 2009 found that 94% were men.^8^ However, it should be noted that more than 90% of urologists are men.^8^ Similar results were found for emergency medicine, where less than 30% of awards were given to women between 2014 and 2018 despite the fact that women make up a much greater share of the physician workforce of emergency medicine compared to urology.^9^ The above-mentioned studies reporting underrepresentation of women as presidents and in board positions of national societies as well as in editorial boards together with our present results complete the picture of a vicious circle of worldwide systematic disadvantage for women in academic medicine. Interestingly, despite the fact that female representation in boards of national nephrology societies was comparably high, the proportion of female award recipients in nephrology was rather low.^6^ This finding was accentuated in recipients of special awards such as the International Society of Nephrology Pioneer Awards.

In conclusion, the current finding that women appear underrepresented as awardees from large, international nephrology societies suggests a substantial gender gap in academic medicine. In contrast, the substantial representation of women among recipients of midcareer awards suggests that progress may be occurring, albeit slowly. Measures to foster women’s careers in medicine are urgently needed, and training and mentorship programs should specifically address gender as role models can inspire female physicians and scientists.

## References

[1] Ryser B., Ravioli S., Lindner G. (2021). Gender distribution in boards of internal medicine societies. Eur J Intern Med.

[2] Ravioli S., Moser N., Ryser B., Pfortmueller C.A., Lindner G. (2022). Gender distribution in boards of intensive care medicine societies. J Crit Care.

[3] Ravioli S., Rupp A., Exadaktylos A.K., Lindner G. (2021). Gender distribution in emergency medicine journals: editorial board memberships in top-ranked academic journals. Eur J Emerg Med.

[4] Ryser B., Rudenko A., Haidinger M., Exadaktylos A.K., Ravioli S., Lindner G. (2022). Gender distribution in speakers at emergency medicine conferences. Am J Emerg Med.

[5] Wiler J.L., Wendel S.K., Rounds K., McGowan B., Baird J. (2022). Salary disparities based on gender in academic emergency medicine leadership. Acad Emerg Med.

[6] Ravioli S., Lindner G., Haidinger M. (2022). Gender distribution in presidents and board members of European nephrology societies. Clin Kidney J.

[7] Malieckal D.A., Ng J.H., Shah H.H., Hong S., Jhaveri K.D. (2020). Trends in the proportions of women program chairs, moderators, and speakers at American Society of Nephrology Kidney Week 2011-2019. Clin J Am Soc Nephrol.

[8] Woldegerima N., Thomopulos A., Bafford A., Malik R.D. (2020). Gender differences in urology society award recipients. Am J Surg.

[9] Krzyzaniak S.M., Gottlieb M., Parsons M., Rocca N., Chan T.M. (2019). What emergency medicine rewards: is there implicit gender bias in national awards?. Ann Emerg Med.

